# Artificial Intelligence in Primary Care Decision-Making: Survey of Healthcare Professionals in Saudi Arabia

**DOI:** 10.7759/cureus.81960

**Published:** 2025-04-09

**Authors:** Najlaa Alsudairy, Alaa Alahdal, Mona Alrashidi, Deemah Altashkandi, Sarah Alzaidi, Afnan Alghamdi, Saud Alzahrani

**Affiliations:** 1 Family Medicine, National Guard Hospital, Jeddah, SAU; 2 Family Medicine, King Abdulaziz University, Jeddah, SAU

**Keywords:** ai adoption, ai tools, artificial intelligence, barriers to adoption, clinical decision-making, decision-making, diagnostic support, healthcare professionals, primary care, training

## Abstract

Background: Artificial intelligence (AI) has the potential to revolutionize healthcare, particularly in primary care, by improving clinical decision-making and patient outcomes. AI technologies, such as machine learning and natural language processing, can assist clinicians in diagnosing conditions, predicting outcomes, recommending treatments, and identifying at-risk individuals. Despite its potential, AI adoption in primary care is slow due to various challenges, including resource limitations, clinician training, and concerns about the reliability of AI systems. Understanding healthcare professionals' perceptions of AI is crucial for overcoming these barriers and promoting its integration into clinical practice.

Methods: A cross-sectional, survey-based study was conducted to assess healthcare professionals' awareness, usage, perceptions, and barriers to AI adoption in primary care decision-making in Saudi Arabia. The study included 250 healthcare professionals from primary care settings across urban, rural, and hospital-based clinics. Data were collected via an electronic survey that included both quantitative and qualitative questions, and analyzed using descriptive and inferential statistics.

Results: A total of 250 healthcare professionals participated in the survey. The majority were primary care physicians (44.8%), with the remaining participants consisting of nurses (27.2%), medical assistants (15.6%), and healthcare administrators (8.8%). Awareness of AI tools was mixed, with 14.8% of respondents very familiar with AI and 47.2% unfamiliar. Thirty-one percent of respondents reported using AI tools, primarily for diagnostic support (59.5%). Common barriers to AI adoption included high implementation costs (49.2%) and lack of training (34%). A significant portion of respondents (48%) expressed concerns about AI undermining the human touch in healthcare.

Conclusions: AI adoption in primary care is hindered by low familiarity and usage, as well as several barriers, including cost, lack of training, and concerns about the reliability of AI systems. However, there is optimism about AI’s potential to support clinical decision-making. Overcoming these barriers through targeted education, infrastructure investment, and further research is essential for promoting AI integration in primary care and realizing its potential benefits for clinical practice.

## Introduction

Artificial intelligence (AI) is transforming healthcare, with the potential to improve clinical decision-making and patient outcomes. AI includes machine learning, natural language processing, and deep learning algorithms, which can analyze vast amounts of data to make predictions and assist in decision-making [1-4]. AI applications in healthcare range from diagnostic imaging and predictive analytics to personalized medicine and treatment recommendations. Integrating AI into clinical workflows promises to enhance decision-making accuracy, reduce errors, and improve patient care [2,5].

AI has the potential to impact primary care by assisting clinicians in diagnosing conditions, predicting outcomes, recommending treatments, and identifying at-risk individuals. AI tools are already being used in diagnostic imaging and to analyze electronic health records for predictive insights. These technologies could reduce diagnostic errors and improve treatment effectiveness [6-9]. However, the adoption of AI in primary care has been slow due to resource limitations, clinician training, and resistance to change. The fragmented nature of primary care and concerns about reliability and errors also pose barriers [5,10].

Understanding healthcare professionals' perceptions of AI is crucial for addressing these adoption challenges. While there is enthusiasm for AI's potential, many clinicians remain cautious about trusting AI without human oversight. Barriers to adoption include financial constraints, lack of training, and concerns about data privacy and security [1-6]. These challenges highlight the need for interventions that address these issues and promote AI integration into primary care.

Education and training are key to overcoming these barriers. Healthcare professionals need to understand how AI works, its limitations, and how to interpret its recommendations. Training programs are being integrated into medical and nursing curricula and ongoing professional development initiatives, which are essential for increasing clinicians' comfort with AI tools and improving adoption rates [5,9,11].

Despite the growing interest in AI's potential in healthcare, there is a significant knowledge gap regarding its practical application in primary care settings. While AI technologies are advancing rapidly, there remains a lack of comprehensive understanding among healthcare professionals about how to effectively integrate these tools into their daily practice [7-10]. Many clinicians are unsure of AI's real-world effectiveness in improving patient outcomes, and there is limited research on its impact in primary care environments. Additionally, the variability in training, resources, and access to AI tools further exacerbates this gap. Future research should focus on closing these gaps by exploring the barriers to AI adoption, evaluating its practical application, and assessing its long-term impact on primary care decision-making and patient care.

## Materials and methods

Study design

This was a cross-sectional, survey-based study aimed at assessing the awareness, usage, perceptions, and barriers associated with the adoption of AI in primary care decision-making. The study was conducted among healthcare professionals working in primary care settings in Saudi Arabia. The survey was designed to collect both quantitative and qualitative data through a structured questionnaire.

Study population

The study population consisted of healthcare professionals, including primary care physicians, nurses, medical assistants, and healthcare administrators, working in urban, rural, and hospital-based primary care clinics in Saudi Arabia. The target sample size was 250 respondents, based on power analysis and estimated response rates, which provided sufficient power for the statistical analyses. Inclusion criteria included healthcare professionals who were actively involved in clinical decision-making in primary care settings. Healthcare professionals who were not involved in direct patient care or were not working in primary care settings were excluded from the study.

Survey development

The survey instrument was developed following an extensive review of existing literature on AI in healthcare decision-making and previous studies on technology adoption in clinical settings. It was structured into four main sections. The first section, Demographic Information, collected data on the respondent’s age, gender, role, years of experience, practice setting, and geographical location. The second section, AI Awareness and Usage, assessed familiarity with AI tools in primary care, along with the frequency and types of AI tools used in clinical practice. The third section, Perceptions of AI in Decision-Making, focused on respondents’ comfort levels with AI in clinical decision-making, their perceptions of AI’s potential benefits, and concerns about its integration into practice. The final section, Barriers to AI Adoption, explored the financial, educational, regulatory, and technological challenges to AI adoption, along with future outlooks on AI’s role in primary care. The questionnaire included multiple-choice and Likert scale questions to measure agreement or disagreement on various statements. It was pre-tested with a small sample of healthcare professionals (n=10) to ensure clarity, validity, and reliability, with minor adjustments made based on their feedback before the full-scale survey was distributed.

Data collection

The survey was distributed electronically via email and through online platforms such as SurveyMonkey, which ensured anonymity and confidentiality of the respondents. The survey link was sent to healthcare professionals working in primary care clinics across Saudi Arabia. A reminder email was sent to non-respondents two weeks after the initial survey distribution. The data collection period lasted for four weeks, from February to March 2025.

Data analysis

Data were analyzed using descriptive and inferential statistics. Descriptive statistics were used to summarize the demographic characteristics of the respondents and the responses to the survey questions. Percentages were calculated for categorical variables (e.g., role, gender, AI familiarity). All analyses were conducted using SPSS Version 28.0 (IBM Corp, Armonk, NY).

Ethical Considerations

This study adhered to ethical guidelines to ensure the protection of respondents' rights. Informed consent was obtained from all participants prior to survey completion. Participants were assured that their responses would remain confidential and anonymous, and that participation was voluntary with the option to withdraw at any time without penalty. No personal identifiers were collected, and data were stored securely.

## Results

Demographic and practice characteristics of survey respondents

A total of 250 healthcare professionals participated in the survey. The majority of respondents were primary care physicians (44.8%, n=112), followed by nurses (27.2%, n=68) and medical assistants (15.6%, n=39). The remaining respondents included healthcare administrators (8.8%, n=22) and other roles (3.6%, n=9). The age distribution of respondents varied, with the largest group being aged 35-44 years (25.2%, n=63), followed by those aged 45-54 years (21.2%, n=53). A smaller proportion of respondents were aged 65 years or older (9.6%, n=24). The gender distribution was nearly equal, with 54.8% (n=137) identifying as male and 45.2% (n=113) identifying as female. Most respondents (60.4%, n=151) worked in urban clinics, while 19.6% (n=49) worked in rural clinics, and 15.6% (n=39) worked in hospital-based primary care settings, as shown in Table 1.

**Table 1:** Demographic and practice characteristics of survey respondents (N=250) Data presented as number (n) and percentage (%).

Characteristic		N (%)
Primary Role in Healthcare Setting	Primary Care Physician	112 (44.8%)
	Nurse	68 (27.2%)
	Medical Assistant	39 (15.6%)
	Healthcare Administrator	22 (8.8%)
	Other	9 (3.6%)
Age Group	18–24 years	16 (6.4%)
	25–34 years	42 (16.8%)
	35–44 years	63 (25.2%)
	45–54 years	53 (21.2%)
	55–64 years	52 (20.8%)
	65 or older	24 (9.6%)
Gender	Male	137 (54.8%)
	Female	113 (45.2%)
Years of Experience	Less than 5 years	35 (14%)
	5–10 years	72 (28.8%)
	11–20 years	81 (32.4%)
	More than 20 years	62 (24.8%)
Type of Healthcare Setting	Urban Clinic	151 (60.4%)
	Rural Clinic	49 (19.6%)
	Hospital-based Primary Care	39 (15.6%)
	Other	11 (4.4%)

AI awareness and usage in primary care

Awareness of AI-based tools for clinical decision-making was mixed. Only 14.8% (n=37) of respondents reported being very familiar with AI tools, while 38% (n=95) were somewhat familiar. The remaining 47.2% (n=118) were not familiar with AI tools. Regarding the usage of AI tools in clinical practice, 31.6% (n=79) of respondents reported having used AI tools. Among these, the most commonly used AI tools were for diagnostic support (59.5%, n=47 of users), followed by treatment recommendations (34.2%, n=27 of users) and patient monitoring and follow-up (10.1%, n=8 of users). Of the respondents who had not used AI tools, the most common reasons cited were lack of awareness or knowledge (42.1%, n=72), lack of resources (23.4%, n=40), and uncertainty about AI reliability (17%, n=29), as shown in Table 2.

**Table 2:** AI awareness and usage in primary care (N=250) Data presented as number (n) and percentage (%).

AI Awareness and Usage		N (%)
Familiarity with AI-based Tools for Clinical Decision-Making	Very familiar	37 (14.8%)
	Somewhat familiar	95 (38%)
	Not familiar at all	118 (47.2%)
Have Used AI Tools in Clinical Practice	Yes	79 (31.6%)
	No	171 (68.4%)
AI Tools Used in Practice	AI for diagnosis support	47 (59.5% of users)
	AI for treatment recommendations	27 (34.2% of users)
	AI for patient monitoring and follow-up	8 (10.1% of users)
Primary Reason for Not Using AI Tools	Lack of awareness or knowledge	72 (42.1%)
	Lack of resources (technology, budget)	40 (23.4%)
	Uncertainty about AI reliability	29 (17%)
	Ethical concerns	19 (11.1%)
	Not applicable (I use AI tools)	11 (6.4%)

Perceptions of AI in primary care decision-making

Respondents exhibited varied comfort levels with using AI-based tools in clinical decision-making. A substantial proportion, 28.8% (n=72), expressed neutrality regarding AI usage, while 22.8% (n=57) felt somewhat uncomfortable and 12% (n=30) felt very uncomfortable. In contrast, only 11.6% (n=29) of respondents were very comfortable using AI tools. Regarding the potential impact of AI on clinical decision-making, 30.4% (n=76) of respondents believed that AI could improve decision-making to some extent, while 39.6% (n=99) disagreed, stating that AI would not improve decision-making. Notably, 19.6% (n=49) of respondents were unsure about the role of AI in improving decision-making. Concerns regarding AI integration into clinical practice were also prevalent, with 48% (n=120) of respondents fearing a loss of human touch, 26% (n=65) expressing a lack of trust in AI recommendations, and 19.2% (n=48) citing concerns about data privacy and security, as presented in Table 3.

**Table 3:** AI awareness and usage in primary care (N=250) Data presented as number (n) and percentage (%).

AI Awareness and Usage		N (%)
Familiarity with AI-based Tools for Clinical Decision-Making	Very familiar	37 (14.8%)
	Somewhat familiar	95 (38%)
	Not familiar at all	118 (47.2%)
Have Used AI Tools in Clinical Practice	Yes	79 (31.6%)
	No	171 (68.4%)
AI Tools Used in Practice	AI for diagnosis support	47 (59.5% of users)
	AI for treatment recommendations	27 (34.2% of users)
	AI for patient monitoring and follow-up	8 (10.1% of users)
Primary Reason for Not Using AI Tools	Lack of awareness or knowledge	72 (42.1%)
	Lack of resources (technology, budget)	40 (23.4%)
	Uncertainty about AI reliability	29 (17%)
	Ethical concerns	19 (11.1%)
	Not applicable (I use AI tools)	11 (6.4%)

Barriers to AI adoption and future outlook

The main barriers to adopting AI in primary care were high implementation costs (49.2%, n=123) and a lack of training or education (34%, n=85). Other significant barriers included resistance from healthcare providers (20.8%, n=52) and regulatory or legal concerns (17.6%, n=44). When asked about the likelihood of recommending AI tools to colleagues, 29.2% (n=73) of respondents were unlikely to recommend them, while 8.8% (n=22) were very likely to do so. Regarding the future role of AI in primary care, 42.4% (n=106) of respondents believed that AI would not have a significant role, while 38% (n=95) felt that it would complement human decision-making. A small proportion (7.6%, n=19) anticipated that AI would become an essential component of primary care in the future, as shown in Table 4.

**Table 4:** Barriers to AI adoption and future outlook (N=250) Data presented as number (n) and percentage (%).

Barriers and Future Outlook		N (%)
Main Barriers to Adopting AI	High implementation costs	123 (49.2%)
	Lack of training and education	85 (34%)
	Resistance from healthcare providers	52 (20.8%)
	Regulatory and legal concerns	44 (17.6%)
	Lack of robust evidence supporting its efficacy	34 (13.6%)
Likelihood of Recommending AI Tools to Colleagues	Very likely	22 (8.8%)
	Somewhat likely	53 (21.2%)
	Neutral	69 (27.6%)
	Unlikely	73 (29.2%)
	Very unlikely	33 (13.2%)
Future Role of AI in Primary Care	AI will become essential	19 (7.6%)
	AI will be complementary to human decision-making	95 (38%)
	AI will not have a significant role	106 (42.4%)
	Not sure	30 (12%)

## Discussion

This study provides insights into the current state of awareness, usage, perceptions, and barriers to the adoption of AI tools in primary care decision-making. Our findings highlight significant opportunities for integrating AI into primary care, as well as notable challenges that must be addressed to facilitate its wider adoption.

Our results demonstrate a clear gap in familiarity with AI technologies among healthcare professionals in primary care. With only 14.8% of respondents being very familiar with AI tools and 47.2% reporting little to no familiarity, it is evident that awareness and education surrounding AI are major hurdles. This is concerning because familiarity with AI has been shown to be a key factor in its acceptance and utilization. If healthcare providers are unaware of the capabilities and potential benefits of AI tools, they are less likely to integrate them into their practice. This gap highlights the urgent need for educational initiatives and training programs designed to increase healthcare professionals' understanding of AI applications in primary care [9-14].

In terms of actual usage, 31.6% of respondents reported using AI tools in clinical practice, most commonly for diagnostic support (59.5% of users). The relatively low usage rate, despite the growing availability of AI tools, suggests that several factors may be limiting their adoption. Our study found that concerns about AI’s reliability and lack of resources were among the top reasons cited for not using AI tools, which mirrors challenges faced by other healthcare systems in adopting new technologies [10,12].

Regarding the perceptions of AI, while 30.4% of respondents believed AI could improve clinical decision-making, 39.6% disagreed, reflecting skepticism about AI’s role in primary care. This mixed response is consistent with studies showing that while some clinicians are optimistic about the potential of AI to support decision-making, others remain cautious due to concerns about the accuracy of AI recommendations and the potential for errors. The reported concerns about AI undermining the human aspect of care (48%) are particularly noteworthy, as they align with broader debates in healthcare about the importance of maintaining the physician-patient relationship, even as technologies become more integrated into clinical practice [13-16].

Our findings have important implications for the integration of AI in primary care. The relatively low usage of AI tools suggests that despite AI's potential to improve decision-making and efficiency, there are significant barriers to its adoption that need to be addressed. High implementation costs (49.2%) and lack of training (34%) were the most commonly cited obstacles. These barriers are consistent with the literature, where financial constraints and a lack of comprehensive training programs for healthcare professionals have been identified as major challenges to the widespread adoption of AI in healthcare. To overcome these barriers, policymakers and healthcare administrators should prioritize funding for AI infrastructure and training programs to ensure that primary care professionals are equipped with the knowledge and resources to use these tools effectively [5-11].

Furthermore, the skepticism about AI’s impact on clinical decision-making and the concerns about loss of human touch suggest that healthcare providers may require more evidence and reassurance about the efficacy and safety of AI tools before they fully embrace them. Research demonstrating the positive outcomes of AI integration in clinical practice, particularly in primary care settings, could help alleviate these concerns and encourage broader adoption [7,9].

The barriers identified in our study-high costs, lack of training, and resistance from healthcare providers-are well-documented in the literature. AI tools often require significant upfront investment in technology and infrastructure, which may be a challenge for smaller primary care practices or those with limited resources. Additionally, the need for comprehensive training programs cannot be overstated, as healthcare professionals need to feel confident in their ability to use AI tools safely and effectively. Overcoming resistance to AI adoption will also require efforts to involve healthcare professionals in the design and implementation of these tools, ensuring that they are user-friendly and aligned with clinical workflows [2-8].

Limitations

While this study provides valuable insights into the current state of AI adoption in primary care, several limitations must be considered. The sample was limited to healthcare professionals working in primary care, and the results may not be representative of those in other healthcare settings, such as specialty care or hospital-based practices. Additionally, the cross-sectional design of the study prevents us from drawing causal inferences about the factors influencing AI adoption. Future research with longitudinal designs could provide a deeper understanding of how attitudes toward AI evolve as these technologies become more integrated into clinical practice.

## Conclusions

In conclusion, our study reveals a significant gap in the awareness and usage of AI tools among primary care professionals, highlighting the challenges faced in integrating these technologies into clinical practice. A major barrier to AI adoption in primary care is the high implementation costs, which pose a financial strain on healthcare settings, particularly in resource-limited environments. Additionally, the lack of sufficient training and education on how to effectively use AI tools hampers clinicians' confidence in incorporating these technologies into their decision-making processes. Concerns regarding AI’s reliability, including its potential to make errors and undermine the human element of patient care, further complicate its adoption. Despite these challenges, there is a strong sense of optimism among healthcare professionals about the potential of AI to enhance decision-making, improve diagnostic accuracy, and streamline treatment recommendations.
